# Therapeutic Potential of Targeting the Cytochrome P450 Enzymes Using Lopinavir/Ritonavir in Colorectal Cancer: A Study in Monolayers, Spheroids and In Vivo Models

**DOI:** 10.3390/cancers15153939

**Published:** 2023-08-02

**Authors:** Maryam Alaei, Seyedeh Elnaz Nazari, Ghazaleh Pourali, AliReza Asadnia, Mehrdad Moetamani-Ahmadi, Hamid Fiuji, Hamid Tanzadehpanah, Fereshteh Asgharzadeh, Fatemeh Babaei, Fatemeh Khojasteh-Leylakoohi, Ibrahim Saeed Gataa, Mohammad Ali Kiani, Gordon A. Ferns, Alfred King-yin Lam, Seyed Mahdi Hassanian, Majid Khazaei, Elisa Giovannetti, Amir Avan

**Affiliations:** 1 Department of Clinical Biochemistry, Mashhad University of Medical Sciences, Mashhad 13944-91388, Iran; alaeim991@mums.ac.ir (M.A.); hasanianmehrm@mums.ac.ir (S.M.H.); 2 Metabolic Syndrome Research Center, Mashhad University of Medical Sciences, Mashhad 13944-91388, Iran; nazarie971@mums.ac.ir (S.E.N.); avana@mums.ac.ir (G.P.); asadniaa4001@mums.ac.ir (A.A.); mehrdadahmadi45@yahoo.com (M.M.-A.); h.tanzadehpanah@gmail.com (H.T.); asgharzadehyf@mums.ac.ir (F.A.); f.babaimedical@gmail.com (F.B.); fatemekhjst@gmail.com (F.K.-L.); khazaeim@mums.ac.ir (M.K.); 3 Basic Sciences Research Institute, Mashhad University of Medical Sciences, Mashhad 13944-91388, Iran; hamid_fiuji@yahoo.com (H.F.); amir_avan@yahoo.com (M.A.K.); 4 Antimicrobial Resistance Research Center, Mashhad University of Medical Sciences, Mashhad 91779-49367, Iran; 5 College of Medicine, University of Warith Al-Anbiyaa, Karbala 56001, Iraq; ibraheem@uowa.edu.iq; 6 Department of Medical Education, Brighton & Sussex Medical School, Falmer, Brighton, Sussex BN1 9PH, UK; g.ferns@bsms.ac.uk; 7 Pathology, School of Medicine and Dentistry, Gold Coast Campus, Griffith University, Gold Coast, QLD 4222, Australia; a.lam@griffith.edu.au; 8 Department of Medical Oncology, Cancer Center Amsterdam, Amsterdam U.M.C., VU. University Medical Center (VUMC), 1081 HV Amsterdam, The Netherlands; 9 Cancer Pharmacology Lab, AIRC Start Up Unit, Fondazione Pisana per La Scienza, 56124 Pisa, Italy; 10 Faculty of Health, School of Biomedical Sciences, Queensland University of Technology, Brisbane, QLD 4059, Australia

**Keywords:** lopinavir/ritonavir, cytochrome P450, colorectal cancer, tumor growth

## Abstract

**Simple Summary:**

We explored the potential of targeting the enzyme cytochrome P450 (CYP450) in colorectal cancer (CRC) using lopinavir/ritonavir. Various experimental methods in a preclinical phase were employed to assess the effects of lopinavir/ritonavir on CRC. The study found that inhibiting CYP450 reduced cell proliferation, induced cell death, and suppressed cell migration. Lopinavir/ritonavir also inhibited tumor growth and fibrosis. These results suggest that targeting CYP450 with lopinavir/ritonavir has therapeutic potential in CRC and highlight the need for further research in this area.

**Abstract:**

Cytochrome P450 (CYP450) enzyme has been shown to be expressed in colorectal cancer (CRC) and its dysregulation is linked to tumor progression and a poor prognosis. Here we investigated the therapeutic potential of targeting CYP450 using lopinavir/ritonavir in CRC. The integrative systems biology method and RNAseq were utilized to investigate the differential levels of genes associated with patients with colorectal cancer. The antiproliferative activity of lopinavir/ritonavir was evaluated in both monolayer and 3-dimensional (3D) models, followed by wound-healing assays. The effectiveness of targeting CYP450 was examined in a mouse model, followed by histopathological analysis, biochemical tests (MDA, SOD, thiol, and CAT), and RT-PCR. The data of dysregulation expressed genes (DEG) revealed 1268 upregulated and 1074 down-regulated genes in CRC. Among the top-score genes and dysregulated pathways, CYPs were detected and associated with poor prognosis of patients with CRC. Inhibition of CYP450 reduced cell proliferation via modulating survivin, Chop, CYP13a, and induction of cell death, as detected by AnnexinV/PI staining. This agent suppressed the migratory behaviors of cells by induction of E-cadherin. Moreover, lopinavir/ritonavir suppressed tumor growth and fibrosis, which correlated with a reduction in SOD/thiol levels and increased MDA levels. Our findings illustrated the therapeutic potential of targeting the CYP450 using lopinavir/ritonavir in colorectal cancer, supporting future investigations on this novel therapeutic approach for the treatment of CRC.

## 1. Introduction

According to recent estimates, colorectal cancer (CRC) ranks as the third leading cause of cancer-related fatalities [1]. According to estimates, the global incidence of colorectal cancer (CRC) exceeded 1.9 million cases in 2020, resulting in over 930,000 deaths. These numbers varied greatly across regions, with notable differences in incidence and mortality rates. Projections indicate that by 2040, the burden of CRC will continue to rise, with an anticipated 63% increase in new cases per year, reaching 3.2 million, and a 73% increase in annual deaths, totaling 1.6 million [2]. The core modalities currently used for the treatment of primary CRC include surgery, targeted therapy, neoadjuvant radiation, and adjuvant chemotherapy and immunotherapy [3]. Despite improvements in response rates with different therapeutic approaches, chemo- and targeted therapies offer these patients only a minimal improvement in overall survival [4]. The treatment of choice for patients with primary CRC continues to be chemotherapy based on 5-fluorouracil (5-FU) [5]. However, 5-FU is only partially effective, with low response and high resistance rates among patients with advanced CRC [6]. These restrictions have prompted additional research on newly developed alternative or combinational medications to enhance anticancer efficacy while lowering toxicity and resistance.

The cytochrome P450 (CYP) superfamily is responsible for the catalytic oxidation of several substances [7]. Recently, it has been demonstrated that CYP isoforms can have significant impacts on tumor growth and development [8,9,10]. Inhibiting these endogenous CYPs in malignant cells may be a new target for anticancer drugs [8,11,12]. The original purpose of HIV-1 protease inhibitors (HIV-PIs) was to target HIV proteases, but they can also interact with mammalian proteins and inhibit cytochrome P450 [13]. HIV protease inhibitors have shown promise in treating Kaposi’s sarcoma and non-Hodgkin lymphoma [14,15], two cancers that are linked to the virus. These results were first linked to immune restoration and improved control of oncogenic viral infections, but several studies in multiple solid tumors (lung, bowel, thyroid, kidney, head/neck, prostate, pancreas, etc.) [16], lymphoma [17], melanoma [18], multiple myeloma [19], and prostate cancer [20] propose alternative pathways by which PIs exert their anti-neoplastic effects. In the past ten years, several independent preclinical and clinical investigations have demonstrated that HIV-PIs have additional pharmacological features that make them likely to be promising anticancer therapeutic agents. Lopinavir and ritonavir are two protease inhibitors, and the FDA (Food and Drug Administration) in the USA has authorized the combination of them for the treatment of HIV-1 [21,22]. Recently, there have been suggestions that these drugs could be used as treatments for cancer [18]. However, there has been relatively limited investigation into the therapeutic possibilities of lopinavir/ritonavir specifically for colorectal cancer. In light of the link between dysregulation of the CYP450 enzyme and poor prognosis in CRC, we conducted a study to explore the therapeutic potential of targeting CYP450 using lopinavir/ritonavir. Our study also aimed to identify genes and pathways that are dysregulated in CRC, with a particular focus on CYPs. To evaluate the effects of lopinavir/ritonavir, we used both in vitro and in vivo models of CRC, assessing its anti-proliferative and anti-migratory activity, as well as its ability to reduce tumor growth and fibrosis.

## 2. Materials and Methods

### 2.1. Chemicals and Drugs

For this study, lopinavir/ritonavir (200/50 mg) and 5-FU were purchased from Abbott C and Ebewe, respectively. RPMI 1640 medium, DMEM, FBS, streptomycin (50 g/mL), and penicillin (50 IU/mL) were all obtained from Betacell in Tehran, Iran. Sigma-Aldrich provided further chemical lab materials (Zwijndrecht, The Netherlands).

### 2.2. Cell Culture

CT-26 and SW-480 cell lines were obtained and certified by the National Cell Bank of Iran. All of the cultured media consisted of RPMI or DMEM, with 10% FBS and 1% streptomycin/penicillin. The culture condition was maintained at 37 °C with a 5% CO_2_. The cells were checked under a microscope to confirm confluency of 80–90%, then cell passaging was performed using trypsin-EDTA. All procedures were according to ATCC instructions.

### 2.3. Patient Samples

Twenty-two colorectal cancer (CRC) tumors and their respective adjacent normal margins, validated by pathologists, were included in the study. Treatment for all eligible patients was administered at Omid Hospital of MUMS (Mashhad University of Medical Sciences), and the patients had not received chemotherapy before. Their clinicopathological characteristics are described in Supplementary Table S1.

### 2.4. Inhibition of Cell Growth

The antiproliferative effect of lopinavir/ritonavir both alone and in combination with 5-FU was estimated via MTT assay after 24 h treatment. CT-26 and SW-480 cells were incubated in a 96-well plate and treated with various concentrations of lopinavir/ritonavir (1–500 μM) as well as its combination with 5-FU at a constant IC50 concentration of 5-FU. Then, the MTT test was assessed [23].

### 2.5. Evaluation of Drug Activity in Multicellular Spheroids

Firstly, 5 × 10^3^ CT-26 cells/mL in a mixture of DMEM/F12 and GlutaMAX-I in a ratio of 1:1 were seeded in 96-well agarose-coated plates to produce multicellular spheroids. After spheroid formation, cells were treated with the IC50 dose in the complete medium for 10 days. The volume (V) of the spheroid was determined using the geometric mean of its perpendicular diameters (D = (Dmax + Dmin)/2) after the spheroid diameter was measured using ImageJ. V = (4/3) × π(D/2)3 [24].

### 2.6. Assessment of Apoptosis

Cell apoptosis was measured by the AnnexinV/P.I. Apoptosis Detection Kit (MabTag, GmbH, Friesoythe, Germany) after treating the cells with IC50 for 24 h. The BD FACSCalibur (BD Biosciences, San Jose, CA, USA) was employed to investigate the proportion of cells undergoing apoptosis [25].

### 2.7. Analysis of Migration by Wound-Healing Assay

As previously referred to, an in vitro migration experiment was utilized to investigate the ability of 5-FU and lopinavir/ritonavir to stop the migration of CT-26 and SW-480 cells [26]. To perform the assay, cells were cultured in 12-well plates until they reached confluence. After creating a straight line in the cell monolayer using a sterile pipette tip, any detached cells were removed by washing the cells with PBS. Subsequently, the cells were subjected to treatment with lopinavir/ritonavir, 5-FU, and a combination of both of them. Images were captured at the time of exposure initiation (Time 0), as well as at 48 and 72 h following the initiation of exposure, and the percentage of wound closure was quantified using image analysis software.

### 2.8. Gene Expression Measurment with qRT-PCR

Cells were treated with lopinavir/ritonavir at its IC50 value and RNA was isolated from the cells following the instructions provided by the manufacturer (Parstous, Tehran, Iran). RNA quality was assessed using a Nanodrop 2000 spectrophotometer (Thermo Scientific, Waltham, USA), and cDNA was then synthesized by Synthesis Kit (Parstous, Tehran, Iran). Quantitative RT-PCR was performed using specific primers from Macrogene (Macrogene Co., Seoul, Republic of Korea). cDNA amplification was performed using the ABI-PRISM StepOne apparatus (Applied Biosystems, Foster City, CA, USA). The data were normalized to GAPDH by utilizing a cDNA standard curve generated from Quantitative PCR Human Reference RNA (Stratagene, La Jolla, CA, USA), as previously described [27].

### 2.9. In Vivo Studies

Female inbred BALB/c mice were procured from the Pasteur Institute and the study was approved by the local committees on animal experimentation of Mashhad University of Medical Sciences, Mashhad, Iran (IR.MUMS.AEC.1401.076). A suspension comprising 2 × 10^6^ CT26 cells per mouse was injected into the animal’s right flank location. Once the tumor attained a size of 80–100 mm^3^, the mice were divided into four groups: a control (*n* = 8) group, a 5-FU (*n* = 8) group (5 mg/kg, every other day, intraperitoneal injection), a lopinavir/ritonavir (*n* = 6) group (100/25 mg/kg for 5 days per week, orally), and a combination (*n* = 6) group.

During the treatment period, tumor growth was monitored using a digital caliper. The mice were sacrificed after day 14 for macroscopic and histological assessment.

### 2.10. Histopathological Staining

Tumor heart, liver, and kidney samples were preserved in a 10% formalin solution for histological examination. Histological analysis was undertaken on tissue that had been embedded in paraffin and 5–7 μm slices. After deparaffinization, these slices were stained with haematoxylin and eosin (H&E) and trichrome and observed under a light microscope (magnification: ×40).

### 2.11. Oxidative Stress Assessment

Tumor tissues were maintained at −70 °C before the measurement of markers of oxidative/antioxidative status. For this, tissue homogenization was performed on ice by using ice-cold phosphate-buffered saline solution. After centrifuging homogenates at 4 °C for 15 min at 3000–4000 rpm, the supernatants were used to detect oxidative and antioxidative markers.

#### 2.11.1. Malondialdehyde (MDA) Assessment

As a biomarker for lipid peroxidation, MDA was used. It was observed how 2-thiobarbituric acid (TBA) and MDA combine to form a pink complex with a maximum absorbance at 535 nm. Two milliliters of each of the TBA, trichloroacetic acid, and hydrochloric acid solutions were mixed with one milliliter of the sample solution before being heated for 45 min. The solution’s absorbance at 535 nm was then calculated after centrifuging it for 10 min. The concentration was calculated by the formula below: (C [M] = A/1.65 × 105) [28].

#### 2.11.2. Measuring Total Thiol Group Concentration

The total thiol levels were measured using the Ellman method. When sulfhydryl groups bonded to carbon react with Ellman’s reagent, 5,5′-dithiobis(2-nitrobenzoic acid; DTNB), a yellow complex with a peak absorbance at 412 nm, is formed. To measure the absorbance, 50 μL of supernatant from each sample was mixed with 1 mL of EDTA buffer and compared to the absorbance of the buffer alone (A1) at 412 nm. After 15 min, the sample absorbance was measured once more, and A2 was labeled after 20 μL of DTNB solution had been added to A1. DTNB absorbance was used as the blank (B). The following equation was used: = (A2 − A1 − B) × 1.07/0.05 × 13.6 [29].

#### 2.11.3. Determination of Superoxide Dismutase (SOD)

The method of Madesh and Balasubramanian was utilized to measure superoxide dismutase (SOD) activity. The method depends on the dependent suppression of MTT to formazan and SOD generation from pyrogallol auto-oxidation. The process was stopped with the addition of DMSO. The supernatant was added to a 96-well plate. After 5 min, DMSO was introduced, and the plate was subsequently analyzed using a microplate reader at 570 nm. One unit of superoxide dismutase (SOD) activity was defined as the amount of protein necessary to prevent a 50% drop in MTT.

#### 2.11.4. Catalase Activity (CAT)

The solution to be used to measure the catalase activity was prepared by mixing 100 μL of H_2_O_2_ with phosphate buffer (pH = 7) (C buffer). In addition, 650 microliters of pH-7.0 phosphate buffer were used as a solution blank. The cuvette was filled with sample homogenates and C buffer before measurements. A spectrophotometer was used to measure the reduction in absorbance for 5 min at a wavelength of 240 nm.

### 2.12. In Silico Analysis

The orientation of lopinavir/ritonavir in the target proteins’ active site was assessed through MOE web-based. We carried out the data analysis by ChemDraw Ultra 7.0 to draw the structure of lopinavir/ritonavir, which was then subjected to energy minimization by MOE. The crystal structure of proteins was obtained from the RCSB Protein Data Bank. The GBVI/WSA dG scoring method was utilized to evaluate the concluding scores [20,21]. The GBVI/WSA dG scoring method was used to investigate the concluding scores, and the binding free energy of the ligand was estimated from a presented pose. The inhibition constant (Ki) was calculated based on the binding free energy estimated using the GBVI/WSA dG scoring function, according to the equation [∆G = RTLn(Ki)], where T represents the temperature in Kelvin and R is the gas constant. Finally, the pKi was calculated from the binding free energy values at a fixed temperature of 298 K using the equation [log Ki = pKi].

### 2.13. Identification of Genes Dysregulated Expression (DEGs)

We undertook gene expression profiling on 22 cases of colorectal cancer (CRC) with the SRA study ID. We utilized the FeatureCounts package in Linux (ubuntu 20.4 LTS). We then normalized the data and analyzed the dysregulation expressed genes (DEGs) using the DESeq2 package (v.1.20) in R software (v.3.6). Next, we compared two conditions: CRC patients with advanced-stage versus normal cases. We used DESeq2 and ClusterProfiler packages to identify up- and down-regulated genes, which were then analyzed for Gene Set Enrichment using the AnnotationHub package to reveal Disease Ontology, Network of Cancer Gene, Gene Ontology, and KEGG Pathways. In addition, gene ontology, which includes biological processes, molecular function, and cellular components, was analyzed using Enrich r to determine the mutual roles of accumulated genes in the cell. To ensure reliable results, we used special criteria, including *p*-values < 0.05 and Log2-fold change |1.5| >, with statistical significance considered at *p* < 0.05.

### 2.14. Statistical Analysis

Results were expressed as mean ± standard error of the mean (SEM). The graphs were created using Graph-Pad Software version 8 and all of the data were analyzed with SPSS version 20 software. Statistical analyses included one-way ANOVA and Tukey–Kramer tests. A *p*-value of <0.05 was considered significant. At least two separate experiments were carried out in triplicate for each experiment to ensure the reliability of the results.

## 3. Results

### 3.1. Gene Signatures and Impact of CYP450 in CRC

**Figure 1:**
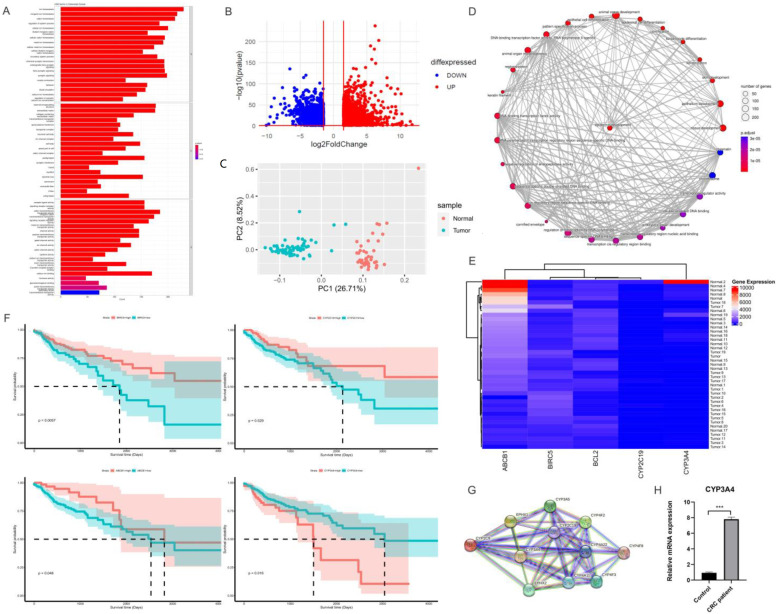
Gene signatures in CRC patients. (**A**–**C**) Molecular function, biological process, and cellular component. (**D**) The gene concept complexities being involved. (**E**) Candidate CYP family enzymes genes and their expression in patients. (**F**) Survival analyses on candidate CYP family enzymes; (**G**) string and network analysis of CYP450 enzymes; (**H**) validation of the impact of CYP3A in an additional cohort of CRC patients as detected by RT-PCR. Bars, SEM. *** *p* < 0.001 different from controls.

After analyzing the dysregulation expressed genes (DEGs), we found that 1268 genes were up-regulated and 1074 genes were down-regulated (Figure 1). The top 40 ranked pathways that were simultaneously activated or suppressed by enriched up- and down-regulated genes in the samples were identified. The data also provided insights into the most related pathways associated with CRC, with a high score for their association with CRC. Among the high top-score genes in the list, CYP450 was associated with a poor prognosis (Figure 1F–G). CYP450 was then evaluated in an additional cohort of CRC patients (Figure 1H) and targeted using FDA-approved lopinavir/ritonavir for further valuation into in vitro and in vivo models of CRC.

### 3.2. Lopinavir/Ritonavir Inhibits Cell Proliferation

**Figure 2:**
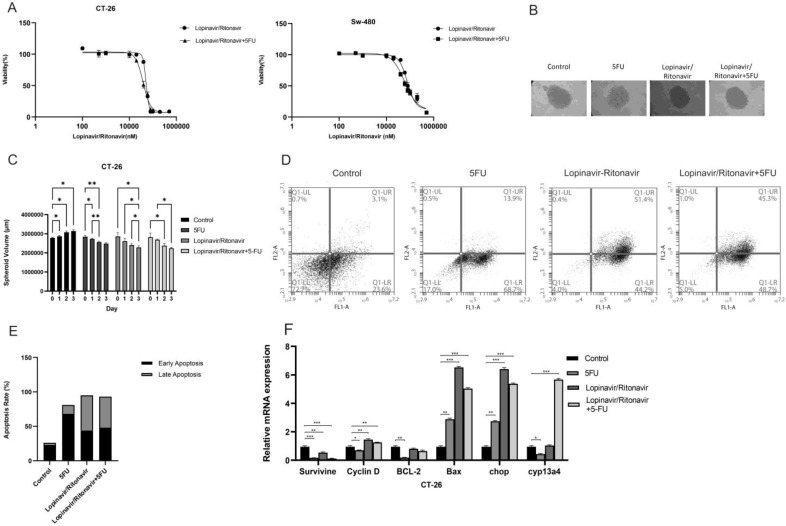
Lopinavir/ritonavir suppresses cell proliferation. (**A**) Growing inhibitory properties of lopinavir/ritonavir in CT-26 and SW-480 cells. (**B**) Results of inhibition of growth in CT-26 spheroids cells. Cells were exposed to lopinavir/ritonavir at IC50 values. (**C**) Spheroid volume after treatment. (**D**) Annexin-V/-FITC/PI analysis of CT-26 colorectal cancer cells treated with 5-FU, lopinavir/ritonavir, and a combination of both. (**E**) Percentage of early and late apoptotic cells after treatment. (**F**) Expression of survivin, CyclinD1, Bcl2, Bax, chop and CYP13a4 before and after treatment as detected by RT-PCR. Bars, SEM. * *p* < 0.05, ** *p* < 0.01 and *** *p* < 0.001 different from controls.

The results of the MTT assay showed a dose-dependent inhibitory activity of lopinavir/ritonavir on cell viability with an IC50 of 51.52 μM and 77.17 μM in CT-26 and SW-480 cells, respectively. Moreover, combining lopinavir/ritonavir with 5-FU had IC50 values of 38.77 μM and 59.24 μM in CT-26 and SW-480 cells (Figure 2A). The inhibitory effect in spheroids was assessed via the 3D model of the CT-26 cell line (Figure 2B). A significant volume decrease in the cultured spheroids was observed in the groups treated with lopinavir/ritonavir relative to the control group (Figure 2C). The Annexin-V/-FITC/PI analysis was performed on CT-26 colorectal cancer cells treated with 5-fluorouracil (5-FU), lopinavir/ritonavir, and a combination of both. The results showed that treatment with 5-FU resulted in a higher percentage of early apoptotic cells (68.7%) compared to Lopinavir/ritonavir (44.2%) or combination treatment (48.7%) (Figure 2D). The apoptosis rate has been shown in Figure 2E. To further investigate the role of lopinavir/ritonavir on the cell cycle, Cyclin D1, and survivin, expression levels were analyzed using quantitative real-time PCR. As observed in Figure 2F, survivin was down-regulated in CT-26 cells.

### 3.3. Lopinavir/Ritonavir Decreases Migration

**Figure 3:**
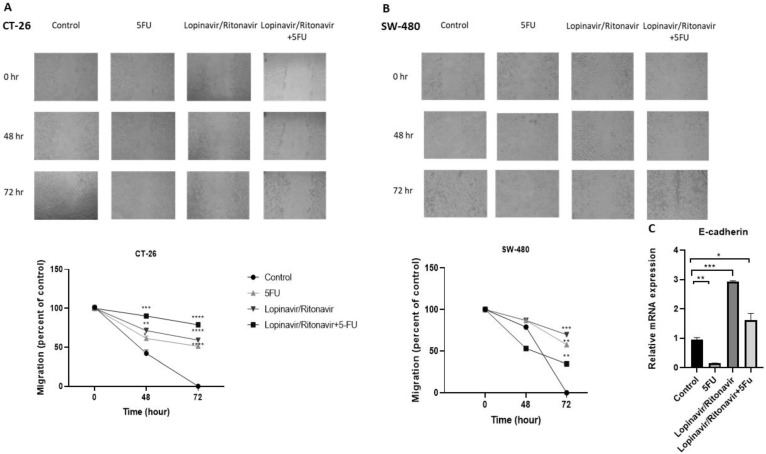
Effects of lopinavir/ritonavir on cell migration. (**A**,**B**) Data of the wound-healing assay in CT-26 and SW-480 cells and (**C**) mRNA expression of E-cadherin. Columns or points, mean values obtained from three independent experiments. * *p* < 0.05, ** *p* < 0.01, *** *p* < 0.001 and **** *p* < 0.0001 compared to positive control.

To examine the inhibitory effect of lopinavir/ritonavir on cell migration, the scratch assay was used. CT-26 and SW-480 cell were treated and evaluated in 0, 48, and 72 h. The results indicated that lopinavir/ritonavir prevents cell migration in both cell lines (Figure 3). To investigate the inhibitory mechanism, the expression level of E-cadherin was measured using qRT-PCR. These data showed a high expression of E-cadherin in lopinavir/ritonavir and combination groups.

### 3.4. Lopinavir/Ritonavir Suppresses Tumor Growth

**Figure 4:**
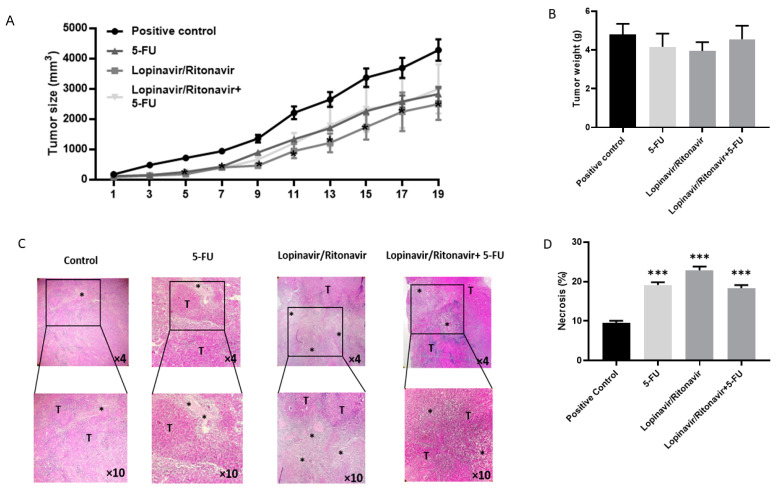
Lopinavir/ritonavir inhibits tumor growth in a mouse model of CRC. (**A**) Tumor size. The mice were divided into four groups: a control group (*n* = 8), a 5-FU group (5 mg/kg, every other day, intraperitoneal injection, *n* = 8), a lopinavir/ritonavir group (100/25 mg/kg for 5 days per week, orally; *n* = 6), and a combination group (*n* = 6). Results were expressed as mean ± standard error of the mean (SEM). * *p* < 0.05, lopinavir/ritonavir compared to control. (**B**) Tumor weight in the CRC mouse model treated with lopinavir/ritonavir, 5-FU, lopinavir/ritonavir + 5-FU. (**C**,**D**) Histological staining of tumor tissue samples by H&E (×10). Tumor tissue exhibited aggregation of tumor cells (T) and necrotic area. Results were expressed as mean ± standard error of the mean (SEM). * *p* < 0.05 and *** *p* < 0.001 compared to positive control.

**Figure 5:**
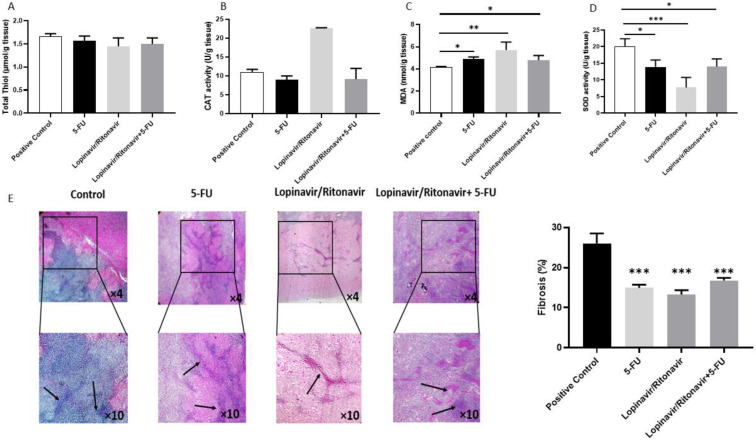
Lopinavir/ritonavir increases oxidative stress and reduces fibrosis. (**A**–**D**) Total thiol, CAT, MDA, and SOD activities in tumor tissues. Columns or points, mean values obtained from three independent experiments; bars, SEM. * Significantly different from controls (* *p* < 0.05, ** *p* < 0.01 and *** *p* < 0.001 compared to positive control). (**E**) Masson’s trichrome staining. Black arrows indicate fibrotic area. * *p* < 0.05, ** *p* < 0.01 and *** *p* < 0.001 compared to positive control.

To further evaluate the results from the in vitro study, an animal model of CRC was developed. As shown in Figure 4, lopinavir/ritonavir and its combination with 5-FU reduced tumor size in comparison with the control group. In a further investigation, the tissue samples were stained using H&E and Masson’s trichrome to assess the value of lopinavir/ritonavir on necrosis and fibrosis. The data demonstrated induced necrosis as well as a reduction in fibrosis (Figure 4 and Figure 5).

### 3.5. The Effect of Lopinavir/Ritonavir on Oxidant/Antioxidant Markers

**Figure 6:**
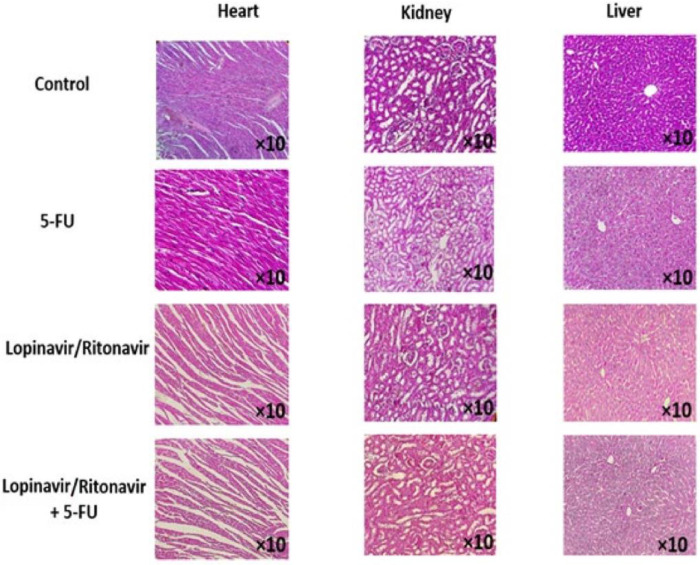
Potential side effects. No significant pathological changes in heart, liver, and kidney were observed in animals following treatment with lopinavir/ritonavir and lopinavir/ritonavir + 5-FU as detected by haematoxylin and eosin staining.

To assess the effect of lopinavir/ritonavir on oxidant/antioxidant balance, we also measured the amounts of thiol, CAT, SOD, and MDA in the homogenized tumor samples. Figure 5 demonstrates a substantial decrease in SOD and thiol content, accompanied by an elevation in MDA levels, in tumor tissue treated with lopinavir/ritonavir or its combination with 5-FU, relative to the control group. Moreover, no significant pathological alterations were observed in the heart, liver, and kidneys of animals after treatment with lopinavir/ritonavir (Figure 6).

**Figure 7:**
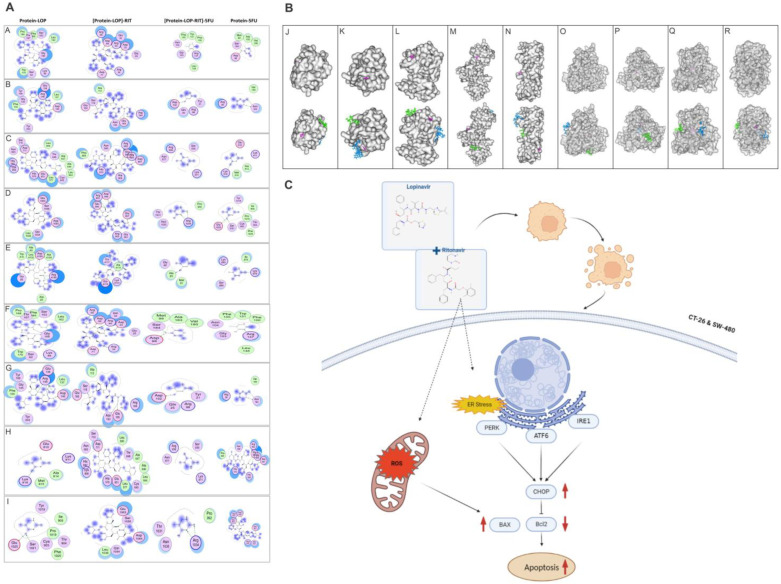
Docking analysis revealed the affinity of lopinavir/ritonavir with target protein CYP450 enzymes. (**A**) Molecular modeling of the interaction between BAX (A), BCL2 (B), PERK (C), IRE1 (D), survivin (E), CYP1A2 (F), CYP2C9 (G), CYP2C19 (H), and CYP3A4 (I) with different ligands. The proteins are shown as surface. The ligands including 5-FU (pink), lopinavir (green), and ritonavir (blue) are shown as sticks. Docking outcomes confirmed that ligands could directly interact with and inhibit CYP450s. (**B**) Molecular modeling of the interaction between BAX (J), BCL2 (K), PERK (L), IRE1 (M), survivin (N), CYP1A2 (O), CYP2C9 (P), CYP2C19 (O), and CYP3A4 (R) with different ligands. Only residues with a distance less than 4.5 Aº to ligands are displayed. (**C**) Schematic illustrates the mechanism of action of lopinavir/ritonavir in the promotion of ER stress to increase apoptosis and reduce cell proliferation in tumor cells.

In addition, we carried out an in-silico analysis (Figure 7) and investigated the affinity of lopinavir/Ritonavir (ligand) with the target proteins BAX, BCL2, PERK, IRE1, survivin, CYP1A2, CYP2C9, CYP2C19, and CYP3A4. The results of docking indicate that ligands could directly interact and inhibit CYP3A4 and CYP2D6 (Score ≈ −7 and −6 kcal/mol, respectively).

Finally, within Table 1, we present the estimated figures for the binding energy between proteins and ligands, along with the amino acids involved in hydrogen bond formation.

## 4. Discussion

We have presented the first proof-of-concept preclinical finding on the potential therapeutic value of lopinavir/ritonavir in the treatment of CRC. The activation and detoxification of a wide range of chemotherapeutic agents are mediated by cytochrome P450 (CYP) enzymes belonging to families one to three [30]. CYP3A4, the most prevalent isoform of the CYP enzyme expressed in the human liver and gut, accounts for 30–60% of total CYPs and plays a crucial role in the metabolism of many chemotherapeutic agents [7,31,32]. Endogenous CYP isoforms that are produced in tumor cells may cause the active medicine to be metabolized into an inactive or less powerful form, changing the therapeutic molecule’s half-life and kinetics. It has been demonstrated that the activity of the CYP3A4 enzyme in CRC cells may affect the tumor’s receptivity to some colon cancer treatments [33]. The current investigation indicated that CYP3A4 expression levels were notably elevated in CRC patients. Elevated expression of CYP3A4 in colorectal cancer patients can result in the greater metabolism of drugs that are metabolized by this enzyme, leading to rapid clearance of the drugs from the body. This can ultimately lead to reduced drug efficacy and potentially hinder the effectiveness of cancer treatments. HIV protease inhibitors (HIV-PIs) have a very wide range of activities and can, in a dose-dependent way, prevent the growth of nearly every cell line examined and/or result in its death [14].

The cytotoxic effects of HIV-PIs have stimulated research on the repurposing of these drugs as anticancer medications [34]. Less research has been done on the effectiveness of lopinavir alone or in conjunction with other medications, and the majority of the findings in this respect are connected to nelfinavir. Ritonavir, saquinavir, and nelfinavir were three of the HIV-PIs that Gills et al. discovered suppressed the proliferation of more than 60 cancer cell lines [35,36]. Lopinavir suppresses meningioma development by causing cell cycle arrest, according to a Johnson et al. study of the drug’s effects on primary cultures of meningioma cells from tumors of various grades [37]. Lopinavir induced caspase-dependent apoptosis and reduced NF-κB activity in primary effusion lymphoma, and its effectiveness was also seen in a xenograft mice model [17]. In renal cells, lopinavir and another protease inhibitor, ritonavir, have demonstrated synergistic antitumor action [38]. Lopinavir, when combined with ritonavir, induced endoplasmic reticulum stress and increased drug levels in the bloodstream, thereby sensitizing head and neck cells to irradiation [39]. In cultures of various bladder cancer cell lines, HIV-PIs significantly increased cell death via a similar mechanism [40]. Tricarico et al. showed lopinavir/ritonavir’s ability to induce apoptosis in neuroblastoma cells (SH-SY5Y) by causing mitochondrial damage, an increase in heme oxygenase RNA expression levels, and the production of ROS [41].

The antitumor efficacy of lopinavir/ritonavir was assessed in colon cancer cells. Our results revealed that lopinavir/ritonavir, as well as its combination with 5-FU, exhibited dose-dependent inhibition of cell growth in CT-26 and SW-480 cell lines. Interestingly, these findings were particularly evident in 3D models, which are more representative of tumor architecture.

The levels of cyclin D1 and survivin were also evaluated, and we found that lopinavir/ritonavir treatment resulted in a significant decrease in the mRNA level of survivin, which is in accordance with prior research showing the growth-inhibiting effects of PIs via downregulation of survivin and induction of apoptosis [42]. It has been shown that ritonavir inhibits the growth of lung cancer cells by reducing survivin as an important target [43]. Ritonavir also reduced the survival levels of T-cell leukemia [44]. We also investigated whether this drug affects the migration of colon cancer cells and examined potential underlying mechanisms. We found that treatment of CT-26 and SW-480 cells with lopinavir/ritonavir decreased the migratory behavior of these cells.

Our results are also in line with previous reports demonstrating the anti-migratory effects of PIs in cancer cell lines [45]. The observed increase in E-cadherin mRNA expression, a cell–cell adhesion molecule known to possess anti-invasive properties, may account for this effect [46]. In support of this hypothesis, Perna et al. indicated the migration-suppressing effect of different anti-HIV drugs via increased expression of E-cadherin in ovarian cancer cell lines after treatment [47]. Another investigation supported the role of dolutegravir against migration and invasion in BT-20 cell lines through enhanced expression of E-cadherin [48]. Our findings consistently demonstrate that treatment of CT-26 cancer cells with lopinavir/ritonavir resulted in a significant rise in the mRNA expression of E-cadherin.

Our in vivo studies showed that lopinavir/ritonavir and its combination with 5-FU inhibited the formation of tumor growth. Moreover, the impact of lopinavir/ritonavir on the oxidant/antioxidant balance in tumor tissue was evaluated by quantifying total thiol content, CAT activity, SOD activity, and MDA levels in the samples [49,50,51]. Several previous studies have confirmed the oxidative effects of HIV-PIs in cancer cells [52,53,54,55]. Xiang et al. found that nelfinavir decreased SOD-2 protein levels and enzyme activity in cervical cancer cell lines [54]. Up-regulation of MDA in animal models treated with HIV-PIs has been shown [56]. In line with these observations, our data revealed an increased MDA level and decreased thiol and SOD activities.

## 5. Conclusions

Our study suggests that lopinavir/ritonavir has promising therapeutic potential for the treatment of colorectal cancer. We found that it effectively inhibits cell growth and migration in CRC cell lines. Our findings also suggest that lopinavir/ritonavir may exert its antitumor effects by inducing apoptosis, as well as suppressing the migratory behavior of CRC cells. Further clinical studies are needed to confirm its clinical efficacy and safety.

## Figures and Tables

**Table 1:** Estimated values of the binding energy between proteins and ligands, and amino acids participating in the formation of hydrogen bonds.

Receptors	Complexes	Energies	H-Bond
BAX	BAX-Lopinavir	−8.88	Gly166
	[BAX-Lopinavir]-Ritonavir	−15.63	Asp33, Arg34, Arg64, Arg65, and Asp68
	[BAX-Lopinavir-Ritonavir]-5FU	−8.02	Asp 98 and Ser184
	BAX-5FU	−8.01	Arg147
BCL2	BCL2-Lopinavir	−11.31	Asp140 and Arg 146
	[BCL2-Lopinavir]-Ritonavir	−17.40	Ser116, Asn163, and Glu156
	[BCL2-Lopinavir-Ritonavir]-5FU	−7.94	Asn163
	BCL2-5FU	−8.08	Tyr21 and Asp102
IRE1	IRE1-Lopinavir	−9.48	Asp711
	[IRE1-Lopinavir]-Ritonavir	−17.16	Asp847, Glu850, and Arg905
	[IRE1-Lopinavir-Ritonavir]-5FU	−9.70	Lys819
	IRE1-5FU	−7.25	Not Found
PERK	PERK-Lopinavir	−7.15	Ser1058
	[PERK-Lopinavir]-Ritonavir	−14.31	Arg891, Glu893, Glu907, and Asp948
	[PERK-Lopinavir-Ritonavir]-5FU	−8.66	Ser1021 and Glu1025
	PERK-5FU	−7.75	Arg1034
Survivin	Survivin-Lopinavir	−10.85	Thr5, Asp105, and Arg106
	[Survivin-Lopinavir]-Ritonavir	−16.56	Glu116 and Thr117
	[Survivin-Lopinavir-Ritonavir]-5FU	−6.70	Lys78
	Survivin-5FU	−7.31	Ala3
CYP1A2	CYP1A2-Lopinavir	−8.15	Tyr160 and Arg281
	[CYP1A2-Lopinavir]-Ritonavir	−15.97	Asp423 and Ser425
	[CYP1A2-Lopinavir-Ritonavir]-5FU	−8.98	Thr124 and Asp313
	CYP1A2-5FU	−8.81	Thr124 and Asp313
CYP2C9	CYP2C9-Lopinavir	−7.04	Asp49 and Lys52
	[CYP2C9-Lopinavir]-Ritonavir	−15.10	Phe419, Asp349, and Glu438
	[CYP2C9-Lopinavir-Ritonavir]-5FU	−9.64	Thr305 and Pro427
	CYP2C9-5FU	−8.58	Thr305 and Pro427
CYP2C19	CYP2C19-Lopinavir	−7.01	His344, Phe419, and Lys421
	[CYP2C19-Lopinavir]-Ritonavir	−16.19	Glu300 and Thr301
	[CYP2C19-Lopinavir-Ritonavir]-5FU	−8.89	Phe476
	CYP2C19-5FU	−10.00	Ile112 and Val436
CYP3A4	CYP3A4-Lopinavir	−7.17	Lys91 and Lys424
	[CYP3A4-Lopinavir]-Ritonavir	−18.44	Lys173, Asp174, Tyr307, Glu308, and Ser312
	[CYP3A4-Lopinavir-Ritonavir]-5FU	−9.32	Leu482
	CYP3A4-5FU	−7.85	Phe302

## Data Availability

The datasets are available upon request.

## Supplementary Materials

The following supporting information can be downloaded at: https://www.mdpi.com/article/10.3390/cancers15153939/s1, Additional information related to the Vivo Studies (number, age, weight, maintenance conditions of mice), Wound-Healing Assay (drug concentrations, FBS percentage), Spheroid Analysis (days number, drug concentrations), Identification of Differentially Expressed Genes (dbGaP Study Accession number, PCA plots and patients samples), PDB-IDs and inhibition constant (Ki) values of docking analyses [57,58,59]. Table S1: Clinicopathological characteristics of the patients.
