# Correction: Alaei et al. Therapeutic Potential of Targeting the Cytochrome P450 Enzymes Using Lopinavir/Ritonavir in Colorectal Cancer: A Study in Monolayers, Spheroids and In Vivo Models. *Cancers* 2023, *15*, 3939

**DOI:** 10.3390/cancers17020325

**Published:** 2025-01-20

**Authors:** Maryam Alaei, Seyedeh Elnaz Nazari, Ghazaleh Pourali, AliReza Asadnia, Mehrdad Moetamani-Ahmadi, Hamid Fiuji, Hamid Tanzadehpanah, Fereshteh Asgharzadeh, Fatemeh Babaei, Fatemeh Khojasteh-Leylakoohi, Ibrahim Saeed Gataa, Mohammad Ali Kiani, Gordon A. Ferns, Alfred King-yin Lam, Seyed Mahdi Hassanian, Majid Khazaei, Elisa Giovannetti, Amir Avan

**Affiliations:** 1 Department of Clinical Biochemistry, Mashhad University of Medical Sciences, Mashhad 13944-91388, Iran; alaeim991@mums.ac.ir (M.A.); hasanianmehrm@mums.ac.ir (S.M.H.); 2 Metabolic Syndrome Research Center, Mashhad University of Medical Sciences, Mashhad 13944-91388, Iran; nazarie971@mums.ac.ir (S.E.N.); avana@mums.ac.ir (G.P.); asadniaa4001@mums.ac.ir (A.A.); mehrdadahmadi45@yahoo.com (M.M.-A.); h.tanzadehpanah@gmail.com (H.T.); asgharzadehyf@mums.ac.ir (F.A.); f.babaimedical@gmail.com (F.B.); fatemekhjst@gmail.com (F.K.-L.); khazaeim@mums.ac.ir (M.K.); 3 Basic Sciences Research Institute, Mashhad University of Medical Sciences, Mashhad 13944-91388, Iran; hamid_fiuji@yahoo.com (H.F.); amir_avan@yahoo.com (M.A.K.); 4 Antimicrobial Resistance Research Center, Mashhad University of Medical Sciences, Mashhad 91779-49367, Iran; 5 College of Medicine, University of Warith Al-Anbiyaa, Karbala 56001, Iraq; ibraheem@uowa.edu.iq; 6 Department of Medical Education, Brighton & Sussex Medical School, Falmer, Brighton, Sussex BN1 9PH, UK; g.ferns@bsms.ac.uk; 7 Pathology, School of Medicine and Dentistry, Gold Coast Campus, Griffith University, Gold Coast, QLD 4222, Australia; a.lam@griffith.edu.au; 8 Department of Medical Oncology, Cancer Center Amsterdam, Amsterdam U.M.C., VU. University Medical Center (VUMC), 1081 HV Amsterdam, The Netherlands; 9 Cancer Pharmacology Lab, AIRC Start Up Unit, Fondazione Pisana per La Scienza, 56124 Pisa, Italy; 10 Faculty of Health, School of Biomedical Sciences, Queensland University of Technology, Brisbane, QLD 4059, Australia

**Error in Figure and Text**

The authors would like to make a correction to their published paper [1].

The methods and Figure 4A have been corrected to include the data of all the mice of the control/standard (*n* = 8) subgroups, in line with the guidelines of the Ethics Committee.

We recalculated the percentual volumetric tumor growth for all included cases and we now present the corrected results together with the updated figure. The interpretation and discussion of the data are not affected by the recalculated values.

The last three sentences of Section 2.9 and the correct Figure 4 are revised as follows.

Once the tumor attained a size of 80–100 mm^3^, the mice were divided into four groups: a control (*n* = 8) group, a 5-FU (*n* = 8) group (5 mg/kg, every other day, intraperitoneal injection), a lopinavir/ritonavir (*n* = 6) group (100/25 mg/kg for 5 days per week, orally), and a combination (*n* = 6) group.

During the treatment period, tumor growth was monitored using a digital caliper. The mice were sacrificed after day 14 for macroscopic and histological assessment.

**Figure 4:**
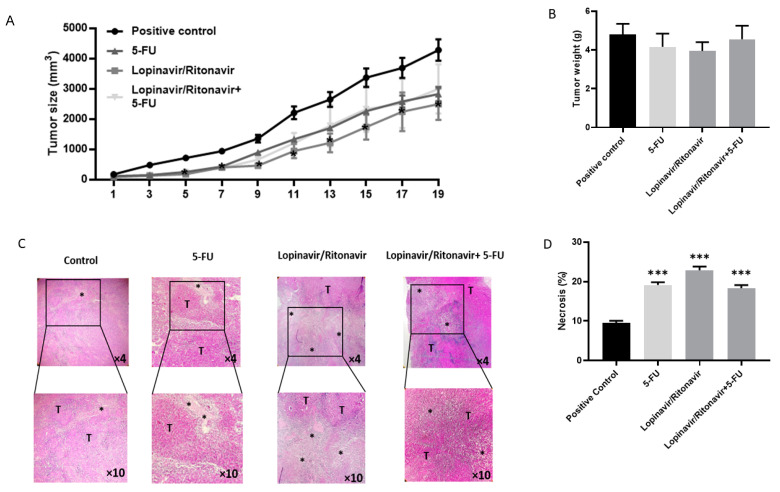
Lopinavir/ritonavir inhibits tumor growth in a mouse model of CRC. (**A**) Tumor size. The mice were divided into four groups: a control group (*n* = 8), a 5-FU group (5 mg/kg, every other day, intraperitoneal injection, *n* = 8), a lopinavir/ritonavir group (100/25 mg/kg for 5 days per week, orally; *n* = 6), and a combination group (*n* = 6). Results were expressed as mean ± standard error of the mean (SEM). * *p* < 0.05, lopinavir/ritonavir compared to control. (**B**) Tumor weight in the CRC mouse model treated with lopinavir/ritonavir, 5-FU, lopinavir/ritonavir + 5-FU. (**C**,**D**) Histological staining of tumor tissue samples by H&E (×10). Tumor tissue exhibited aggregation of tumor cells (T) and necrotic area. Results were expressed as mean ± standard error of the mean (SEM). * *p* < 0.05 and *** *p* < 0.001 compared to positive control.

**Modify Supplementary Materials**

Additional information related to the Vivo Studies (number, age, weight, maintenance conditions of mice), as well as to Wound-Healing Assay (drug concentrations, FBS percentage), Spheroid Analysis (days number, drug concentrations), Identification of Differentially Expressed Genes (dbGaP Study Accession number, PCA plots and patients samples), PDB-IDs and inhibition constant (Ki) values of docking analyses, have been incorporated into the Supplemental Information. At the same time, the new references are also added to the original text.

The authors apologize for any inconvenience caused and state that the scientific conclusions are unaffected. This correction was approved by the Academic Editor. The original article has been updated.
