# Supplementary material for: Therapeutic Potential of Targeting the Cytochrome P450 Enzymes Using Lopinavir/Ritonavir in Colorectal Cancer: A Study in Monolayers, Spheroids and In Vivo Models
Source: Cancers (Basel). 2023 Aug 2;15(15):3939. doi: 10.3390/cancers15153939 (PMC10417395; doi:10.3390/cancers15153939)
Supplement: Supplementary file 1 [file cancers-15-03939-s001.zip › cancers-2510145-supplementary/cancers-2510145-supplementary.pdf]

Supplemental Methods - Information

In Vivo Studies

Animal studies were carried out based on the Mashhad University of Medical Science's Animal Experimentation Ethics Committee. Pasteur Institute provided eight-week-old female inbred BALB/c mice with an average weight of 20–22 gr (Tehran, Iran). CT-26 cells ($2*10^6$) were subcutaneously inserted into the right flank of each mouse. Once the tumor attained a size of 80–100 mm^3, the mice were divided into four groups: a control (n=8) group, a 5-FU (n=8) group (5 mg/kg, every other day, intraperitoneal injection), a lopinavir/ritonavir (n=6) group (100/25 mg/kg for 5 days per week, orally), and a combination (n=6) group, as reported in previous studies (IR.MUMS.MEDICAL.REC.1400.700). During the treatment period, tumor growth was monitored using a digital caliper. The mice were sacrificed on day 14 for macroscopic and histological assessment (magnification: ×4 and ×10). The experiment was performed under animal ethical code of IR.MUMS.AEC.1401.076, as reported in the specific document:

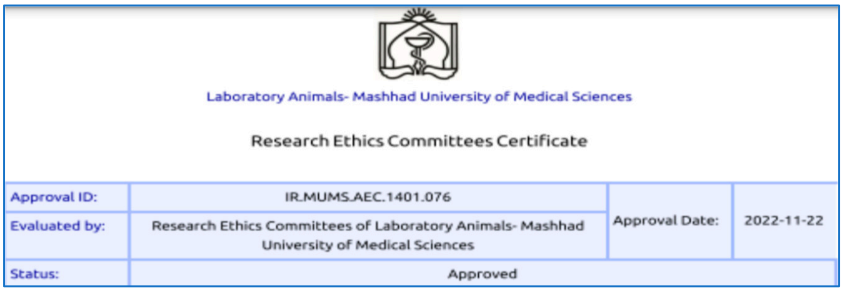

Laboratory Animals- Mashhad University of Medical Sciences

Research Ethics Committees Certificate

Approval ID:	IR.MUMS.AEC.1401.076	Approval Date:	2022-11-22
Evaluated by:	Research Ethics Committees of Laboratory Animals- Mashhad University of Medical Sciences		
Status:	Approved		

The following studies on Histopathological Staining, Oxidative Stress Assessment, Malondialdehyde (MDA) Assessment, Measuring Total Thiol Group Concentration, Determination of Superoxide Dismutase (SOD) and Catalase Activity (CAT) were performed in representative groups of 5-6 animals, as described previously (*Jamialahmadi H, Nazari SE, TanzadehPanah H, et al. Targeting transforming growth factor beta (TGF-β) using Pirfenidone, a potential repurposing therapeutic strategy in colorectal cancer. Sci Rep. 2023 Sep 1;13(1):14357. doi: 10.1038/s41598- 023-41550-2. PMID: 37658230; Ellman GL. Tissue sulfhydryl groups Arch Biochem Biophys.1959 May;82(1):70-7. doi: 10.1016/0003-9861(59)90090-6; Madesh M, Balasubramanian KA. Microtiter plate assay for superoxide dismutase using MTT reduction by superoxide. Indian J Biochem Biophys. 1998 Jun;35(3):184-8. PMID: 9803669*). However, tumor weight and necrosis were also analyzed in groups of n=8 mice for both control and 5-FU-treated animals, as reported in Fig. S1 and S2, respectively.

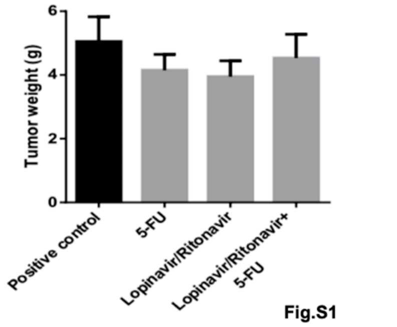

Fig.S1

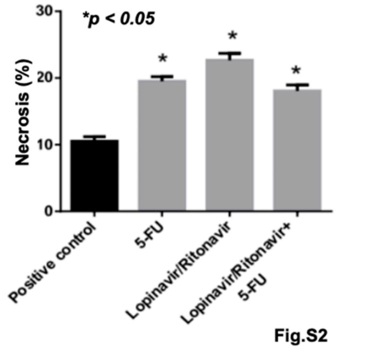

Fig.S2

The concentration of MDA was calculated using the formula: $C [M] = A/1.65 \times 10^5$. We also checked that the statistical difference between lopinavir/ritonavir treated groups versus control for CAT activity was $p < 0.05$.

Wound-Healing Assay

An in vitro migration experiment was conducted to investigate the effects of 5-FU and lopinavir/ritonavir on cell migration. Briefly, the cells were cultured in 12-well plates until they reached confluence. A straight-line scratch was then created in the cell monolayer using a sterile pipette tip. Detached cells were removed by washing with PBS, and the remaining cells were treated with lopinavir/ritonavir, 5-FU, or their combination at non-toxic concentrations. The medium's FBS concentration was reduced to 1% to limit proliferation. Images were captured at 0, 48, and 72 hours, and the percentage of the wound area was quantified using ImageJ software. The results, presented in *Figure 3A-B*, show the percentage of wound area at different time points.

Spheroid Analysis

The spheroid volume unit was expressed in μm^3, with the X-axis values of 0, 1, 2, and 3 corresponding to days 0, 3, 6, and 9, respectively. The concentrations of lopinavir/ritonavir are reported in nM, as shown in *Figure 2A/C*.

Identification of Differentially Expressed Genes (DEGs)

The RNA-seq data for colorectal cancer (CRC) were sourced from The Cancer Genome Atlas (TCGA) dataset, accessible through the Genomic Data Commons (GDC) Data Portal at www.portal.gdc.cancer.gov.

The dbGaP Study Accession number for the dataset used is phs000178.

Two conditions: CRC patients with advanced-stage versus normal cases were compared. Normalization was carried out, while the PCA plots (data from 111 samples, consisting of 70 metastatic colorectal cancer patients and 41 normal controls), volcano plots and heatmap were assessed by the R packages "ggplot2", and "ComplexHeatmap" to visualize data. Significance analysis of differentially expressed genes (DEGs) was performed using DESeq2 in R software with the cutoff criteria of |log fold change | ≥ 1.5 and an adjusted p-value of <0.05. We used DESeq2 packages to identify up- and down-regulated genes. In addition, gene ontology, which includes biological processes, molecular function, and cellular components, was analyzed using ClusterProfiler packages. In particular, the significant enrichment analysis of DEGs was assessed based on Gene Ontology (GO). GO analysis (http://www.geneontology.org/) was used for annotating genes and gene products and investigating the biological aspects of high-throughput genome or transcriptome data, including biological processes, cellular components, and molecular function. The STRING database (https://string-db.org/) was used to investigate the relationship between CYP450 enzymes. For the survival analysis, we utilized the Kaplan-Meier method, implemented using the "survival" and "survminer" packages in R version 4.2.1 software. These packages are well-established tools for survival analysis and visualization in the R programming environment. Then, the candidate gene was validated in 22 cases of colorectal cancer (CRC) by RT-PCR (*Figure 1H*).

Inhibition constant (Ki) values

Receptors (PDB ID)	Complexes	Binding Energies (kcal/mol)	Inhibition Constant (Ki value)	*p*ki
BAX (5W62)	BAX-Lopinavir	-8.88	0.3	6.5
	[BAX-Lopinavir]-Ritonavir	-15.63	3.4×10^{-6}	11.5
	[BAX-Lopinavir-Ritonavir]-5FU	-8.02	1.31	5.9
	BAX-5FU	-8.01	1.33	5.9
BCL2	BCL2-Lopinavir	-11.31	0.005	8.3

(6GL8)	[BCL2-Lopinavir]-Ritonavir	-17.40	1.73×10^{-7}	12.8
	[BCL2-Lopinavir-Ritonavir]-5FU	-7.94	1.5	5.8
	BCL2-5FU	-8.08	1.18	5.9
IRE1 (4U6R)	IRE1-Lopinavir	-9.48	0.11	6.9
	[IRE1-Lopinavir]-Ritonavir	-17.16	2.5×10^{-7}	12.6
	[IRE1-Lopinavir-Ritonavir]-5FU	-9.70	0.08	7.1
	IRE1-5FU	-7.25	4.81	5.3
PERK (4G31)	PERK-Lopinavir	-7.15	5.7	5.2
	[PERK-Lopinavir]-Ritonavir	-14.31	3.19×10^{-5}	10.5
	[PERK-Lopinavir-Ritonavir]-5FU	-8.66	0.44	6.3
	PERK-5FU	-7.75	2.06	5.7
Survivin (1XOX)	Survivin-Lopinavir	-10.85	0.01	7.9
	[Survivin-Lopinavir]-Ritonavir	-16.56	7.15×10^{-7}	12.1
	[Survivin-Lopinavir-Ritonavir]-5FU	-6.70	12.2	4.9
	Survivin-5FU	-7.31	4.34	5.4
CYP1A2 (2HI4)	CYP1A2-Lopinavir	-8.15	1.05	5.9
	[CYP1A2-Lopinavir]-Ritonavir	-15.97	1.94×10^{-6}	11.7
	[CYP1A2-Lopinavir-Ritonavir]-5FU	-8.98	0.25	6.6
	CYP1A2-5FU	-8.81	0.34	6.5
CYP2C9 (1OG2)	CYP2C9-Lopinavir	-7.04	6.86	5.1
	[CYP2C9-Lopinavir]-Ritonavir	-15.10	8.41×10^{-6}	11.1
	[CYP2C9-Lopinavir-Ritonavir]-5FU	-9.64	0.08	7.1
	CYP2C9-5FU	-8.58	0.5	6.3
CYP2C19 (4GQS)	CYP2C19-Lopinavir	-7.01	7.2	5.1
	[CYP2C19-Lopinavir]-Ritonavir	-16.19	1.33×10^{-6}	11.9
	[CYP2C19-Lopinavir-Ritonavir]-5FU	-8.89	0.3	6.5
	CYP2C19-5FU	-10.00	0.04	7.3
CYP3A4 (4D6Z)	CYP3A4-Lopinavir	-7.17	5.5	5.2
	[CYP3A4-Lopinavir]-Ritonavir	-18.44	2.9×10^{-8}	13.5
	[CYP3A4-Lopinavir-Ritonavir]-5FU	-9.32	0.14	6.8
	CYP3A4-5FU	-7.85	1.74	5.7

The orientation of lopinavir/ritonavir in the target proteins' active site was assessed through MOE web-based (1-3). We carried out the data analysis by ChemDraw Ultra 7.0 to draw the structure of lopinavir/ritonavir, which was then subjected to energy minimization by MOE. The crystal structure of proteins was obtained from the RCSB Protein Data Bank. The inhibition constant (Ki) was calculated based on the binding free energy estimated using the GBVI/WSA dG scoring function, according to the equation [$\Delta G = RTLn(Ki)$], where T represents the temperature in Kelvin and R is the gas constant. Finally, the pKi was calculated from the binding free energy values at a fixed temperature of 298 K using the equation [log Ki = pKi].

1. Kharazmi-Khorassani J, Asoodeh A, Tanzadehpanah H. Antioxidant and angiotensin-converting enzyme (ACE) inhibitory activity of thymosin alpha-1 (Thα1) peptide. Bioorg Chem. 2019 Jun;87:743-752. doi: 10.1016/j.bioorg.2019.04.003. Epub 2019 Apr 4. doi: 10.1016/j.bioorg.2019.04.003PMID: 30974297.

2. Tanzadehpanah H, Bahmani A, Hosseinpour Moghadam N, et al. Synthesis, anticancer activity, and β-lactoglobulin binding interactions of multitargeted kinase inhibitor sorafenib tosylate (SORt) using spectroscopic and molecular modelling approaches. Luminescence. 2021; 36: 117–128. https://doi.org/10.1002/bio.3929. PMID: 32725773

3. Morris GM, Goodsell DS, Huey R, Olson AJ. Distributed automated docking of flexible ligands to proteins: parallel applications of AutoDock 2.4. J Comput Aided Mol Des. 1996 Aug;10(4):293-304. doi: 10.1007/BF00124499. PMID: 8877701.

Table S1. Clinicopathological characteristics

Clinicopathological Variables	No. of patients (%) /mean ± SD
Patients	22
Mean age (Years, mean ± SD)	58.61 ± 1.34
Sex	
Male	12 (54.5)
Female	10 (45.5)
Grade	
Poorly Differentiated	2 (9.0)
Moderately Differentiated	14 (63.6)
Well Differentiated	6 (27.2)
Depth of tumor invasion (T)	
T1	0
T2	5 (22.7)
T3	12 (54.5)
T4	2 (9.0)
Lymph node involvement (N)	
Yes	16 (72.7)
No	6 (27.2)
Metastasis (M)	
Yes	8 (36.5)
No	14 (63.3)
Family history	
Yes	2 (6.6)
No	28 (93.3)
